# Development and validation across trimester of the Prenatal Eating Behaviors Screening tool

**DOI:** 10.1007/s00737-022-01230-y

**Published:** 2022-05-02

**Authors:** Elizabeth A. Claydon, Christa L. Lilly, Jordan X. Ceglar, Omar F. Dueñas-Garcia

**Affiliations:** 1 Department of Social and Behavioral Sciences, West Virginia University School of Public Health, 64 Medical Center Drive, P.O. Box 9190, Morgantown, WV 26505 USA; 2 Department of Biostatistics, West Virginia University School of Public Health, Morgantown, WV USA; 3 Department of Obstetrics & Gynecology, West Virginia University School of Medicine, Morgantown, WV USA

**Keywords:** Pregnancy, Psychometrics, Mental health, Feeding and eating disorders, Pregnancy trimesters, Reproducibility of results, Anorexia nervosa, Bulimia nervosa, Binge eating disorder

## Abstract

Rapid screening tools are useful for identifying at-risk patients and referring them for further assessment and treatment, but none exist that consider the unique medical needs of pregnant women with eating disorders (EDs). There is a need for a rapid, sensitive, and specific screening tool that can be used to identify a potential ED in pregnancy. We started with a set of 25 questions, developed from our qualitative work along with other ED screening tools, and tested on a development (*n* = 190) and validation sample (*n* = 167). Statistical analysis included factor analysis and logistic regressions with ROC curves. Development and validation samples were combined for trimester analysis (*n* = 357). Refining the tool to 12 items demonstrated strong internal reliability (development alpha = 0.95, validation alpha = 0.91). With correlated errors, questions demonstrated acceptable CFA fit (development: GFI: 0.91, RMSEA: 0.10, NNFI: 0.95; validation: GFI: 0.85, RMSEA: 0.14, NNFI: 0.86). Similar fits were seen by trimester: first trimester *n* = 127, GFI: 0.89, RMSEA: 0.12, NNFI: 0.94; second trimester *n* = 150, GFI: 0.83, RMSEA: 0.14, NNFI: 0.88; third trimester *n* = 80, GFI: 0.99, NNFI: 0.99. Validation against current ED diagnosis demonstrated acceptable sensitivity and specificity using a cutoff of 39 (development sensitivity = 80.7%, specificity = 79.7%, OR = 16.42, 95% CI: 7.51, 35.88; validation sensitivity = 69.2%, specificity = 86.5%, OR: 17.43, 95% CI: 6.46, 47.01). Findings suggest the PEBS tool can reliably and sensitively detect EDs across pregnancy trimesters with 12 questions. A further implication of this work is to reduce health and mental health treatment disparities through this standard and rapid screening measure to ensure early identification and treatment.

**Supplementary Information:**

The online version contains supplementary material available at 10.1007/s00737-022-01230-y.

## Introduction

The impact that eating disorders (EDs) have in pregnancy is considerable, although the best way to identify these disorders during this unique time remains to be determined. Estimates suggest that at least 5% of women experience some type of ED during pregnancy (Linna et al. 2014; Watson et al. 2013), although some estimates are much broader from 0.6 to 27.8% (Broussard 2012; Bye et al. 2020; Easter et al. 2013; Micali et al. 2007; Pettersson et al. 2016; Soares et al. 2009). These estimates reflect some of the challenges in identifying an ED in this population.

Women with EDs are more likely to have unplanned pregnancies, miscarriages, nutritional differences, and an increased risk of postpartum depression and anxiety as opposed to women without eating disorders (Chan et al. 2019; Kimmel et al. 2015; Zerwas & Claydon 2014). Some women with past EDs have similar nutritional patterns during pregnancy as individuals without EDs and many experiences an improvement in nutrition with pregnancy (Dörsam et al. 2019). However, there are some nutritional differences among individuals with a past or active ED which could affect fetal development. Additionally, there is burgeoning epigenetic research to show how modification of the genome can occur prenatally due to malnourishment, which can affect the developing fetus initially and throughout their life (Hoffman et al. 2017; Sebastiani et al. 2020). Identifying women with a current ED, those who are at-risk for developing an ED, and/or those who are at risk to relapse during pregnancy provides an opportunity for early intervention to improve outcomes for the mother and developing fetus.

Among women with infertility, 58% of those who presented with oligomenorrhea or amenorrhea had some form of an ED, none of whom disclosed those to their providers (Bruneau et al., 2017; Linna et al. 2014). This is consistent with recent qualitative evidence which suggests that many pregnant women with EDs during pregnancy or with a history of an ED do not feel comfortable disclosing information about their ED with their healthcare providers (Claydon et al., 2018). Other qualitative work on this topic indicates several barriers for identifying EDs during pregnancy which reinforce issues of stigma as well as limits to professional training (Bye et al. 2018). Additionally, most medical professionals are not well-trained in identifying pregnant women who may have an ED or clinically significant ED symptomatology unless they are in the psychiatry discipline or have taken continuing medical education specific to EDs (Anderson et al. 2017; Leddy et al. 2009). There is minimal training on EDs for medical professionals outside of the psychiatry specialty (Mahr et al., 2014), leaving most medical professionals who interact with pregnant women with limited knowledge on how to identify and help these patients.

To fill the gap with identification, rapid screening tools can be useful to assist with early screening. These rapid screening tools can assist in identifying at-risk patients, but currently, there are none that consider the unique characteristics and situation of pregnant women with EDs. The SCOFF is a five-item simple clinical tool used to identify a potential case of anorexia nervosa (AN) or bulimia nervosa (BN), but it is not validated in pregnancy (Morgan et al. 2013). Recent research has suggested that when used in a pregnant population, it can provide false positives, which has resulted in an expert consensus that the SCOFF is not the ideal tool for use in these populations (Bannatyne et al. 2018; Baudet et al. 2013). Currently, the Eating Disorder Examination Questionnaire (EDE-Q; Fairburn 2008) has the most psychometric data available in pregnancy but needs further validation and is too lengthy with 28 questions (Bannatyne et al. 2019).

Given those concerns, a few researchers have tried to adapt current measures such as the SCOFF (Hubin-Gayte and Squires 2012) or the EDE (Bannatyne et al. 2018) for use in a pregnant population. One revised version of the SCOFF used in a French pregnant population tried separating the SCOFF questions into pre-pregnancy and during pregnancy categories to identify and understand antenatal ED symptoms (Hubin-Gayte and Squires 2012). However, this method does not truly capture ED symptoms accurately during pregnancy (the time when both mother and developing fetus are at risk), and limited psychometrics were reported in the study. The adaptation of the EDE for pregnancy, known as the EDE-PV, had adequate internal reliability (0.59–0.67 for subscales) in a population of pregnant women who were overweight and/or obese (Bannatyne et al. 2018; Emery et al. 2017). However, the EDE-PV is a structured interview with multiple sections and is better for use as a full assessment, rather than for quick clinical identification. The EDE-PV has also not been validated in pregnant women who are under a BMI of 30 (Emery et al. 2017). But the use of BMI during pregnancy has been a topic of debate and it appears that is more accurate in the first trimester as the pregnancy progresses there are some other physiologic changes that may affect this parameter (Louise et al. 2020).

The aim of this study was to determine the psychometric properties for the Prenatal Eating Behaviors Screening (PEBS) Tool, which was created to address these concerns. The goal of this tool was to be rapid (less than 5 min to complete), applicable to most common EDs in pregnancy [AN, BN, binge eating disorder (BED), and other specified feeding and eating disorders (OSFED)], and valid across the span of pregnancy (all three trimesters). This was accomplished using large validation and development samples and making use of experts in both medical and ED research fields. Once validation was shown, the goal is for the Prenatal Eating Behaviors Screening (PEBS) Tool to be used as a rapid screen tool to identify pregnant women at risk in order to refer them for a specialist for further assessment.

## Methods

### PEBS item development

A series of questions were included from existing sources (e.g., EDE-Q, Fairburn 2008; SCOFF, Morgan et al. 1999; EDI-3, Clausen et al. 2011) and from pilot data themes from a previous study (Claydon et al. 2018). An initial list of 34 questions were combined into a single framework and reviewed by content experts, with an eye towards (1) adapting or using language to be appropriate for terms that should be used or avoided in the ED field (e.g., using person-first language; Weissman et al. 2016), (2) adapting or using language appropriate for women throughout their pregnancy (e.g., removing items that looked for a certain amount of weight loss over several months), and (3) making items follow similar Likert type patterns of responses for ease of respondent use (e.g., using “you” for stem, checking for consistency in “strongly agree” to “strongly disagree” in responses). Finally, questions were checked to ensure all four types of ED symptomology were represented by a content expert, and then a total of 9 questions were dropped for redundancy reasons. A final set of 25 questions underwent further piloting by a small sample (*n* = 3) of pregnant women with a diagnosed ED or history of an ED to check for face validity, accuracy in wording, and question interpretation by questioning relevance, language sensitivity, and accuracy as they filled out the study; all 25 items were then included in the development and validation samples. Supplemental Table 1 has the full list of 25 items along with a number of women responding to each Likert-type response for both samples.

#### Self-report ED diagnoses

As part of the survey, participants were asked to self-report whether they had ever been diagnosed with an eating disorder. If they selected yes, they were asked “Which eating disorders have you had” and could select any of the following: AN, BED, BN, or OSFED, formerly known as eating disorders not otherwise specified (EDNOS). Participants responded to each of those prompts by selecting: current professional diagnosis, past professional diagnosis, current self-diagnosis, or past self-diagnosis (see Appendix 1 for the complete survey). They were allowed to choose more than one answer in each category. Self-report ED diagnoses have been shown to be adequate in detecting current and lifetime AN and BN as measured by diagnostic interviews (Keski-Rahkonen et al. 2006). Participants were counted as having a current diagnosis (yes) if they selected either current professional diagnosis and/or current self-diagnosis for any of the four ED types. These current self-reported diagnoses were used because it was not feasible for the scope of a psychometric study to conduct full diagnostic interviews on all participants.

#### Pregnancy trimester

Pregnancy trimester was assessed by a self-reported week of pregnancy and categorized as first trimester, weeks 4–13; second trimester, weeks 14–27; and third trimester, weeks 28–40.

### Participants

Two separate samples were collected for this study. The development sample was collected first via Amazon Mechanical Turk (MTurk, an online crowdsourcing site to complete tasks virtually), and online mother and pregnancy groups with an IRB-approved advertisement (see Appendix 2). This included 271 responses recorded between August 2020 and November 2020. Next, the validation sample was collected via SurveyCircle (an online survey exchange platform), a second MTurk sample excluding “super users”, and online mother and pregnancy groups and included 236 responses recorded between October 2020 and March 2021. Sample collection methods differed slightly based on including additional exclusions in MTurk sampling following recommendations from Litman and Robinson (2021) and then extending sampling to SurveyCircle to allow for additional responses in the validation sample. All participants were recruited from online groups based in North America or the UK; SurveyCircle has geographic constraints which were enabled to similarly draw from participants in North America and the UK. Although we did not ask about geographic location, other studies suggest the majority of users on MTurk are from the USA (75%) with India second at 16%, and Canada third at 1.1% (Difallah et al., 2018; Moss et al. 2020). A final restriction of English-language proficiency to complete this survey suggests that the majority of the sample are likely located in North America and the UK.

#### Human subjects

This study was filed with the referent university’s Institutional Review Board and exempt status was acknowledged (IRB#: 2003925385). Qualtrics software was used to host and distribute the survey and no protected health information (PHI) was obtained.

#### Data quality checks

Both datasets underwent a series of data quality checks to eliminate possible “bot” submissions and other data quality problems. First, cases were eliminated if they did not list the weeks pregnant as a number between 4 and 40 (development *n* = 67, validation *n* = 55). The weeks pregnant were selected as the initial restriction as it is a sample requirement that only pregnant women be included. This pregnancy week selection eliminated those not pregnant, unsure about pregnant status, and probable “bot” included but unlikely values (e.g., 80). Next, cases were eliminated if they did not complete at least 90% of the survey (development *n* = 9, validation *n* = 9). This was part of the decision tree because those cases would be dropped via pairwise deletion if they did not fill out the final 25 survey questions related to the eating disorder tool. Finally, cases were eliminated if they completed the survey in under 2 min (development *n* = 5, validation *n* = 5); this is assuming a loss of comprehension when reading speed exceeded 15 words per second. Final datasets included *n* = 190 (70.1%) for development, and *n* = 167 (70.8%) for validation. These response rates after quality control are consistent, if not higher with what is being seen for MTurk and other online survey platforms (Kennedy et al. 2020; Nayak and Narayan 2019). All analyses were conducted with SAS software, version 9.4 (SAS Institute, Inc. 2013).

## Results

### Development

Analyses were conducted first on the development dataset (*n* = 190). The goal of the developmental dataset was data reduction (to reduce from 25 items to a minimum number of items to accurately screen pregnant women for an ED) with a focus on relationship with current ED diagnosis. Within the development sample, the majority were 25–34 years old (*n* = 104; 54.74%), married (*n* = 164; 86.32%), White (*n* = 140; 74.07%), and had private insurance (*n* = 129; 67.89%). The mean week of pregnancy was 19.26 (SD = 10.61), and 30.0% had a current ED diagnosis (*n* = 57). Full descriptive statistics and demographic characteristics of the sample are presented in Table 1.

**Table 1:** Demographics and descriptive statistics for development (*n* = 190) and validation (*n* = 167) samples

	Development	Validation
Variable	*M* or *n*(SD or %)	*M* or *n*(SD or %)
Current week of pregnancy	19.26 (10.61)	18.19 (9.06)
Age		
18–24	17 (8.95)	43 (25.75)
25–34	104 (54.74)	91 (54.49)
35–44	60 (31.58)	27 (16.17)
45 +	9 (4.74)	6 (3.59)
Relationship status		
Married/other^‡^	164 (86.32)	96 (57.49)
Singled/widowed	5 (2.63)	17 (10.18)
Living with partner	12 (6.32)	36 (21.56)
In a relationship	9 (4.74)	18 (10.78)
Race		
White	140 (74.07)	107 (64.07)
Black	6 (3.17)	20 (11.98)
Asian	39 (20.63)	30 (17.96)
Other	4 (2.12)	10 (5.99)
Hispanic of Latino descent		
Yes	44 (23.78)	18 (10.84)
No	141 (76.22)	148 (89.16)
Number of children		
0 children	30 (15.79)	61 (36.53)
1 child	96 (50.53)	69 (41.32)
2 children	50 (26.32)	28 (16.77)
3 + children	14 (7.37)	9 (5.39)
Number of pregnancies (including current)		
1	41 (21.58)	78 (46.71)
2	85 (44.74)	51 (30.54)
3	44 (23.16)	22 (13.17)
4	11 (5.79)	6 (3.59)
5 +	9 (4.74)	10 (5.99)
Average total household annual income		
Less than $19 k	19 (10.00)	25 (14.97)
$20 k–$49 k	58 (30.53)	55 (32.93)
$50 k–$149 k	70 (36.84)	66 (39.52)
$150 k–$249 k	30 (15.79)	12 (7.19)
$250 k +	12 (6.32)	3 (1.80)
Prefer not to answer	1	6 (3.59)
Working status		
Student	6 (3.16)	43 (25.75)
Unemployment, retired/disability	13 (6.84)	11 (6.59)
Part-time	43 (22.63)	31 (18.56)
Full-time	126 (66.32)	75 (44.91)
Prefer not to answer	2 (1.05)	7 (4.19)
Type of medical insurance		
Private	129 (67.89)	90 (53.89)
State (Medicaid)	51 (26.84)	43 (25.75)
None	5 (2.63)	17 (10.18)
Prefer not to answer	5 (2.63)	17 (10.18)
Previous eating disorder diagnosis		
Yes	72 (37.89)	44 (26.35)
No	118 (62.11)	123 (73.65)
Eating disorders		
Anorexia nervosa		
None	127 (66.84)	130 (77.84)
Current professional Dx	9 (4.74)	5 (2.99)
Past professional Dx	16 (8.42)	11 (6.59)
Current self Dx	26 (13.68)	8 (4.79)
Past self Dx	12 (6.32)	13 (7.78)
Bulimia nervosa		
None	123 (64.74)	134 (80.24)
Current professional Dx	9 (4.74)	3 (1.80)
Past professional Dx	21 (11.05)	10 (5.99)
Current self Dx	15 (7.89)	8 (4.79)
Past self Dx	22 (11.58)	12 (7.19)
Binge eating disorder		
None	126 (66.32)	133 (79.64)
Current professional Dx	6 (3.16)	3 (1.80)
Past professional Dx	15 (7.89)	11 (6.59)
Current self Dx	31 (16.32)	9 (5.39)
Past self Dx	12 (6.32)	11 (6.59)
Other specified feeding and eating disorder		
None	134 (70.53)	143 (85.63)
Current professional Dx	4 (2.11)	-
Past professional Dx	14 (7.37)	4 (2.40)
Current self Dx	15 (7.89)	8 (4.79)
Past self Dx	23 (12.11)	12 (7.19)
Eating disorder duration		
0	118 (62.11)	123 (73.65)
< 1 year	20 (10.53)	18 (10.78)
1–3 years	29 (15.26)	11 (6.59)
4–6 years	16 (8.42)	7 (4.19)
7 + years	7 (3.69)	5 (4.79)
Current ED dx (professional/self)		
Yes	57 (30.00)	26 (15.57)
No	133 (70.00)	141 (84.43)
Currently seeking treatment or counseling for an eating disorder		
None	118 (62.11)	123 (73.65)
Seeking counseling	33 (17.37)	18 (10.78)
Seeking in-pt	18 (9.47)	7 (4.19)
Not seeking	21 (11.05)	19 (11.38)
Weight change during current pregnancy		
Lost more than 15lbs	4 (2.19)	3 (2.14)
Lost 14–10lbs	4 (2.19)	4 (2.86)
Lost 9–5lbs	6 (3.28)	8 (5.71)
Stayed the same	8 (4.37)	3 (2.14)
Gained less than 5lbs	20 (10.93)	17 (12.14)
Gained 5–10lbs	48 (26.23)	31 (22.14)
Gained 10lbs	37 (20.22)	34 (24.29)
Gained 15lbs	18 (9.84)	16 (11.43)
Gained 20lbs	18 (9.84)	10 (7.14)
Gained 30lbs	10 (5.46)	5 (3.57)
Gained more than 40lbs	10 (5.47)	9 (6.43)
Highest completed level of education		
Grade school/high school/GED	11 (5.79)	29 (17.37)
Vocational school	7 (3.68)	2 (1.20)
College	97 (51.05)	71 (42.51)
Professional/graduate school	75 (39.47)	65 (38.92)

^‡^Includes divorced and separated

The Development sample

#### Internal reliability

Cronbach’s alpha was used to ensure the 25 items were consistent and had internal reliability; item correlations below 0.60 were first considered as possible for deletion. Five items were below 0.20, and another three below 0.60.

#### Relationship with current ED diagnosis

Each of the 25 items was then examined using non-parametric Mann–Whitney tests against self-reported current ED diagnosis (Table 2). The five items with the lowest internal consistency also did not have a statistically significant relationship with current ED diagnosis, alpha set to 0.05. Two items with modest internal reliability (below 0.60) had statistically significant but more modest relationships (*p*-values between 0.002 and 0.005) relative to the other items. All other items *p* < 0.0001.

**Table 2:** Items content with the single factor EFA solution on the polychoric correlation matrix, development only (*n* = 190), of pattern coefficients, *p*-values for differences between current diagnosis, and other goodness-of-fit indicators

Expert content analysis	Item #	Item question	Factor loadings all items	Factor loadings, 12 items	Mann–Whitney *p*-value, current ED dx
General	Q1	How comfortable are you with being weighed today by a health professional?	0.00	–	0.49
General	Q2	Are you satisfied with your pregnancy weight gain?	− 0.13	–	0.10
General	Q3	During this pregnancy, how satisfied are you with your weight progression?	− 0.20	–	0.35
General	Q4	During this pregnancy, how satisfied are you currently seeing your own body in a mirror, while undressing, etc.?	− 0.16	–	0.11
General	Q5	During this pregnancy, how satisfied are you with your body's appearance generally?	− 0.12	–	0.33
Bulimia nervosa	Q6	During this pregnancy, how frequently, if at all, have you used any pregnancy symptoms to control weight? (e.g., morning sickness, nausea, etc.)	0.81	0.80	< 0.0001
Bulimia nervosa	Q7	During this pregnancy, how frequently, if at all, have you used diuretics, laxatives, or detox supplements to control your weight or shape in response to food intake? (e.g., probiotics, metabolism boosters, Lasix, etc.)	0.92	0.94	< 0.0001
Bulimia nervosa	Q8	During this pregnancy, how frequently, if at all, have you made yourself sick after eating in order to control your weight or shape?	0.92	0.93	< 0.0001
Bulimia nervosa	Q9	During this pregnancy, how frequently, if at all, did you excessively exercise as a response to food intake? (e.g., to influence weight or shape)	0.87	0.88	< 0.0001
Anorexia nervosa	Q10	During this pregnancy, how frequently, if at all, did you avoid eating any foods which you like in order to influence your shape or weight?	0.74	0.73	< 0.0001
Bulimia nervosa	Q11	During this pregnancy, how frequently, if at all, did you think of trying to vomit in order to lose weight?	0.93	0.93	< 0.0001
Binge eating disorder	Q12	During this pregnancy, how frequently, if at all, did you experience a loss of control in overeating unrelated to pregnancy cravings?	0.83	0.83	< 0.0001
Bulimia nervosa/binge eating disorder	Q13	During this pregnancy, how frequently, if at all, did you go on eating binges where you felt that you could not stop?	0.84	0.83	< 0.0001
Anorexia nervosa	Q14	During this pregnancy, how frequently, if at all, did you spend a majority of your day thinking about food, weight, counting calories, or other weight related topics?	0.69	–	< 0.0001
Anorexia nervosa/binge eating disorder	Q15	During this pregnancy, how frequently, if at all, did you feel you couldn't control what you were eating and/or excessively exercise in order to control your weight?	0.79	0.78	< 0.0001
Anorexia nervosa	Q16	During this pregnancy, how frequently, if at all, did you emphasize the importance of weight? (e.g., to others, to yourself, etc.)	0.69	–	< 0.0001
Anorexia nervosa/general	Q17	During your pregnancy, how frequently, if at all, has thinking about your shape or weight interfered with your ability to concentrate on things?	0.81	0.79	< 0.0001
General	Q18	During this pregnancy, how frequently, if at all, have you felt guilty about eating?	0.64	–	< 0.0001
Anorexia nervosa	Q19	During your pregnancy, how frequently, if at all, have you restricted your portion sizes?	0.73	0.72	< 0.0001
General	Q20	During this pregnancy, you eat much more when you are alone than when you are in front of others	0.62	–	< 0.0001
General	Q21	During this pregnancy, food has dominated your life	0.69	–	< 0.0001
Anorexia nervosa	Q22	During this pregnancy, you have had the desire for your stomach to feel hungry	0.75	0.74	< 0.0001
Anorexia nervosa	Q23	During this pregnancy, you have been fearful of weight gain	0.44	–	0.005
Anorexia nervosa	Q24	During this pregnancy, you have been fearful of losing your pregnancy weight	0.46	–	0.002
Anorexia nervosa	Q25	During this pregnancy, it bothers you when you are weighed (e.g., at the doctor’s office.)	0.61	–	< 0.0001
		Eigenvalue	75.57	44.83	
		Proportion variance explained	0.64	0.94	
		AIC	1553.86	183.55	
		Tucker and Lewis’s reliability coefficient	0.58	0.88	

#### Exploratory factor analysis

Next, a polychoric correlation matrix was output from the 25 items and used for all further factor analyses. First, exploratory factor analysis (EFA) was run with squared multiple correlation (smc) priors and full information maximum likelihood method, with a one and then two factor solution. Single factor with all items demonstrated best fit (Table 2: 64% variability, Tucker and Lewis: 0.58, AIC: 1553.86). Factor loadings below 0.70 were then removed, and another EFA run with a single factor on the final 12 items, resulting in significantly improved model fit (94% variance explained, Tucker and Lewis: 0.88, AIC: 183.55, all factor loadings above 0.72.)

#### Confirmatory factor analysis

EFA results guided CFA models, starting with a single factor solution, and then adding correlated errors of items related by ED diagnosis, and then substituting a second factor for ED diagnosis, all on the polychoric matrix of the items. The best fitting model was the single factor solution with correlated errors and is presented in Table 3. All factor loadings *p* < 0.0001. Final model included eight correlated errors, including three correlated errors pertaining to items related to BN, three correlated errors related to items about BED, and two sets of correlated errors for AN items (see Supplemental Path Diagram Figure A1 for correlated error terms). With correlated errors, questions demonstrated acceptable model fit (e.g., GFI: 0.91, RMSEA: 0.10, NNFI: 0.95).

**Table 3:** Confirmatory factor analysis results for development (*n* = 190) and validation (*n* = 167) samples, and then by trimester across both samples (*n* = 357) for the final 12 item instrument

	Development	Validation	Trimester		
			First	Second	Third^a^
Sample*n*	190	167	127	150	80
Cronbach’s alpha	0.95	0.91	0.95	0.93	0.92
Model fit					
Chi-square df	46	46	46	46	46
Chi-square	131.53	196.41	91.46	177.06	–
Chi-square*p*-value	< 0.0001	< 0.0001	< 0.0001	< 0.0001	–
Standardized root mean square residual	0.03	0.06	0.04	0.07	0.05
GFI	0.91	0.85	0.89	0.83	0.99
RMSEA	0.10	0.14	0.12	0.14	–
90% lower CL RMSEA	0.08	0.12	0.09	0.12	–
90% upper CL RMSEA	0.12	0.16	0.15	0.16	–
AIC	195.53	260.41	179.46	241.06	–
Bentler comparative fit index	0.96	0.90	0.96	0.91	–
Bentler-Bonett NFI	0.95	0.86	0.94	0.88	0.99
Items	Standardized estimates (se)				
Q6	0.81 (0.03)	0.58 (0.05)	0.69 (0.05)	0.71 (0.04)	0.75
Q7	0.94 (0.01)	0.84 (0.03)	0.91 (0.02)	0.91 (0.02)	0.87
Q8	0.95 (0.01)	0.92 (0.02)	0.94 (0.01)	0.93 (0.01)	0.99
Q9	0.88 (0.02)	0.79 (0.03)	0.88 (0.02)	0.79 (0.03)	0.84
Q10	0.74 (0.03)	0.53 (0.06)	0.73 (0.04)	0.56 (0.06)	0.66
Q11	0.93 (0.01)	0.85 (0.02)	0.93 (0.01)	0.88 (0.02)	0.91
Q12	0.80 (0.03)	0.69 (0.04)	0.79 (0.04)	0.74 (0.04)	0.65
Q13	0.77 (0.03)	0.76 (0.04)	0.77 (0.04)	0.69 (0.05)	0.68
Q15	0.75 (0.03)	0.76 (0.04)	0.78 (0.04)	0.69 (0.05)	0.76
Q17	0.73 (0.03)	0.70 (0.04)	0.80 (0.03)	0.74 (0.04)	0.65
Q19	0.70 (0.04)	0.57 (0.05)	0.75 (0.04)	0.57 (0.06)	0.62
Q22	0.73 (0.03)	0.70 (0.04)	0.74 (0.04)	0.69 (0.04)	0.71
Summed score against current ED diagnosis					
*M* (SD)	32.48 (13.9)	28.29 (10.9)	33.70 (14.0)	29.69 (11.7)	27.04 (11.5)
OR (each unit)	1.16	1.16	1.17	1.15	1.15
Wald 95% CL upper	1.11	1.10	1.11	1.09	1.06
Wald 95% CL upper	1.21	1.23	1.23	1.21	1.25
Hosmer and Lemeshow goodness of fit	7.81, *p* = 0.45	5.54, *p* = 0.70	4.95, *p* = 0.67	7.65, p = 0.47	12.82, p = 0.12
AUC	0.88	0.88	0.89	0.85	0.88
Value at which sensitivity and specificity is maximized	39	–	–	–	–
*N*(%) at or above cutoff	73 (38.4%)	65 (38.9%)	62 (53.5%)	53 (32.9%)	22 (27.5%)
Sensitivity	80.7	69.2	77.8	67.9	70.0
Specificity	79.7	86.5	79.3	82.0	85.7
OR at cutoff	16.42	17.43	17.68	13.64	14.00
95% CL upper	7.51	6.46	6.96	5.16	3.10
95% CL upper	35.88	47.01	44.92	36.04	63.32

^a^Third trimester CFA run with ULS method due to small sample size

#### Final scale clinical cutoff score and validation against current ED diagnosis

The final 12-item scale demonstrated excellent internal reliability, Cronbach’s alpha = 0.95. Items were summed and ranged from 12 to 60, *M* = 32.48 (SD = 13.9). Those with current ED diagnosis (*n* = 57, *M* = 45.63, SD = 8.4) had higher scores against all other participants (*n* = 133, *M* = 26.85, SD = 11.9), *t* (df = 188) = 12.37, *p* < 0.0001. Using logistic regression with receiver operating characteristic curve on current ED diagnosis, we found increased odds of 1.16 (Wald 95% CL 1.11, 1.21) for each point increase in the summed score, Table 3. A cutoff of 39 had good sensitivity (80.7%) and specificity (79.7%) for detecting current ED diagnosis, with an AUC of 0.88 (Fig. 1a). Those with a score of 39 or greater had 16.42 increased odds of having a current ED diagnosis relative to those with a score below 39. The final scale can be found in Appendix 3. Lower cutoffs could be used in order to maximize sensitivity over specificity. For example, a cutoff score of 34 gives a sensitivity of 89.5%, but reduces specificity to 71.4%. Using this cutoff results in *N* = 73, 38.4% of the sample as being identified by the screening tool, resulting in a similar OR of 16.4.

### Validation

Final scale reliability and CFA were replicated with the smaller validation sample (*n* = 167), using the same cutoff of 39 found in the developmental dataset. The validation sample looked similar to the development sample. A majority of the sample were 25–34 years old (*n* = 91; 54.49%), married (*n* = 96; 57.49%), White (*n* = 107; 64.07%), and had private insurance (*n* = 90; 53.89%). The mean week of pregnancy was 18.91 (SD = 9.06), and 15.6% had a current ED diagnosis (*n* = 26).

The 12-item scale demonstrated slightly attenuated but still excellent internal reliability, Cronbach’s alpha = 0.91. Model fit statistics of the CFA on the polychoric matrix attenuated (Table 3); three items standardized factor loading dropped below the preferred 0.60 although all *p*-values remained significant. Although some model fit indicators also dropped below preferred values (e.g., RMSEA), generally acceptable model fit was also found for the validation model (i.e., GFI: 0.85, RMSEA: 0.14, NNFI: 0.86). Logistic regression indicated well-fitting sensitivity (69.2%) and specificity (86.5%) with the new sample at the same cutoff value of 39, with an AUC of 0.88 (Fig. 1b).

### Trimester

Next, we wished to ascertain that the model would work for each trimester of pregnancy. Due to small samples within trimesters, the development and validation samples were combined for this analysis and then stratified by trimester. Due to the small sample for the third trimester, a different method (ULS) for the CFA was used, and thus, some estimates are not available. The polychoric correlation matrix by trimester was used for all factor analyses. Generally acceptable model fit indices were found (first trimester *n* = 127, GFI: 0.89, RMSEA: 0.12, NNFI: 0.94; second trimester *n* = 150, GFI: 0.83, RMSEA: 0.14, NNFI: 0.88; third trimester *n* = 80, GFI: 0.99, NNFI: 0.99). AUC against current ED diagnosis ranged from 0.85 to 0.89 (Fig. 2). Sensitivity (67.9 to 77.8%) and specificity (79.3 to 85.7%) were also acceptable with the cutoff score of 39 and retained increased odds of having a current ED diagnosis relative to a score below 39 (OR range: 13.64 to 17.68).

## Discussion

The findings from this research suggest the PEBS tool can reliably and sensitively detect EDs in pregnancy with only 12 questions. This would provide a rapid initial screen to allow clinicians to refer women for further assessment. The American College of Obstetricians and Gynecologists (ACOG) in their practice bulletin 740 states that health care providers should be comfortable screening and recognizing patients with eating disorders. Still, only a professional expert should make the final diagnosis (ACOG 2018). It is well known that patients with eating disorders are at risk of having higher rates of anxiety and postpartum depression and babies with a small fetal head circumference (Kimmel et al. 2016). Universal screening for ED in pregnancy has not been established or recommended by professional organizations, although it has been acknowledged that there are clinical management strategies that are currently missing (Paslakis and Zwaan 2019). Considering that many patients with eating disorders engage in weight cycling, clinicians may focus on those groups during pregnancy (Marchesini et al. 2004). Applying the PEBS tool to those patients at the highest risk, could represent an opportunity for clinicians to identify a potential ED in pregnancy. Identifying EDs during pregnancy may allow for appropriate referrals to mental health providers, physicians, and registered dietitians to improve women’s psychological and physical well-being during pregnancy and promote better maternal and child health outcomes.

### Strengths and limitations

The final PEBS tool with 12 items meets clinical recommendations, suggesting that brief screening instruments contain 15 items or less and use a simple cutoff score (Marquer et al. 2012). This allows the tool to be easily used by clinicians who have limited time with patients. Additionally, this screening tool was tested on a large sample of pregnant women across all trimesters.

There are some limitations inherent in the methodology which are important to note. First, the sample collected were only English-speaking since the tool is designed to be validated in English first. Therefore, the PEBS tool may not be culturally or linguistically appropriate in all populations and will need to be tailored and translated as needed. Second, convenience sampling was employed which has biases based on that sampling technique. Third, the data collection for the development and validation sample was conducted at slightly different time points, although within a few months of each other. Fourth, there was a smaller sample gathered for the third trimester, which gives us less information on how the tool works relative to the first two trimesters, although there is still sound psychometric data for the third trimester. All model fits ranged from acceptable to good. However, future studies may consider more stringent model fit criteria. Fifth, our final alpha coefficients are high (above 0.90), which raises some question about scale homogeneity at the expense of content coverage and validity (Streiner 2003). However, we believe that we addressed this concern by having content experts review the final 12 items to ensure that we incorporated symptomatology in the final questions that demonstrated the multifaceted nature of the breadth of eating disorders. Sixth, we did not conduct diagnostic interviews with participants to gather their current and past ED diagnoses, but instead asked for self-report of self and professional diagnoses. Comparison with a diagnostic tool would be ideal, but self-reported diagnoses have been shown to be a good indicator of current and lifetime ED diagnoses (Keski-Rahkonen et al. 2006). Additionally, survey collection was conducted during the COVID-19 pandemic and for the safety of participants, an online survey was deemed to be most appropriate. One diagnostic online survey assessment possibility is the digital version of the eating disorder assessment for DSM-5 (EDA-5; Sysko et al. 2015). However, the EDA-5 takes approximately 20–30 min to complete, significantly adding to participant burden and reducing the likelihood of obtaining a large enough sample for meaningful results. Finally, the PEBS tool may screen positive for women with a prior ED, but who are currently in recovery. However, the sensitivity and specificity are high for the PEBS tool, which indicates that women are being identified based on their current risk.

## Future research and conclusions

One of the most critical aspects of research is the translation, in order to ensure that the information gathered is made applicable and available to the people that it can help the most. To that end, following this publication, informational booklets on how to use the PEBS tool will be made available to clinicians, along with referral resources. This will bridge the research-practice gap that perpetuates incremental change rather than allowing for broad dissemination of results. Additionally, a feasibility study will be conducted in order to see how the 12 item PEBS tool works in a clinical setting. To address the limitations of self-report diagnoses, this follow-up study will utilize a smaller confirmatory sample with diagnostic interviews for EDs. A pencil/paper and online version of the PEBS tool have also been created to allow for further ease of dissemination and use. Additionally, future research will have to tailor and translate this tool so that it can be more linguistically and culturally appropriate for diverse populations. A further implication of this work is to reduce health and mental health treatment disparities in pregnant women through this standard and rapid screening measure to ensure early identification and treatment. 

## Supplementary Information

Below is the link to the electronic supplementary material.

Supplementary file1 (DOCX 21 KB)

Supplementary file2 (DOCX 36 KB)

Supplementary file3 (PDF 3305 KB)

Supplementary file4 (JPG 293 KB)

Supplementary file5 (PDF 166 KB)

## Data Availability

Data and materials are available upon reasonable request from the authors. The content is solely the responsibility of the authors and does not necessarily represent the official views of the National Institutes of Health.

## References

[CR1] ACOG Committee Opinion No (2018). 740: Gynecologic care for adolescents and young women with eating disorders. Obstet Gynecol.

[CR2] Anderson K, Accurso EC, Kinasz KR, Le Grange D (2017) Residents’ and fellows’ knowledge and attitudes about eating disorders at an academic medical center. Acad Psychiatry 41(3):381–384. 10.1007/s40596-016-0578-z 10.1007/s40596-016-0578-z PMC7219944 27882518

[CR3] Bannatyne AJ, Hughes R, Stapleton P, Watt B, MacKenzie-Shalders K (2018) Signs and symptoms of disordered eating in pregnancy: a Delphi consensus study. BMC Pregnancy and Childbirth 18(1). 10.1186/s12884-018-1849-3 10.1186/s12884-018-1849-3 PMC6019208 29940882

[CR4] Bannatyne, A. J., McNeil, E., Stapleton, P., MacKenzie-Shalders, K., & Watt, B. (2019). Disordered eating measures validated in pregnancy samples: a systematic review. *Eating Disorders*, 1–26. 10.1080/10640266.2019.1663478 10.1080/10640266.2019.1663478 31675283

[CR5] Baudet ML, Montastier E, Mesthe P, Oustric S, Lepage B, Ritz P (2013). The SCOFF score: a screening tool for eating disorders in family practice. e-SPEN Journal 8(3):e86–e89. 10.1016/j.clnme.2013.03.001

[CR6] Broussard B (2012) Psychological and behavioral traits associated with eating disorders and pregnancy: a pilot study. J Midwifery Womens Health 57(1):61–66. 10.1111/j.1542-2011.2011.00089.x 10.1111/j.1542-2011.2011.00089.x 22251914

[CR7] Bruneau M, Colombel A, Mirallié S, Fréour T, Hardouin J-B, Barrière P, Grall-Bronnec M (2017) Desire for a child and eating disorders in women seeking infertility treatment. PLOS ONE 12(6). 10.1371/journal.pone.0178848 10.1371/journal.pone.0178848 PMC5460847 28586392

[CR8] Bye A, Shawe J, Bick D, Easter A, Kash-Macdonald M, Micali N (2018). Barriers to identifying eating disorders in pregnancy and in the postnatal period: a qualitative approach. BMC Pregnancy Childbirth.

[CR9] Bye A, Nath S, Ryan EG, Bick D, Easter A, Howard LM, Micali N (2020). Prevalence and clinical characterisation of pregnant women with eating disorders. European Eating Disorders Review : the Journal of the Eating Disorders Association.

[CR10] Chan CY, Lee AM, Koh YW, Lam SK, Lee CP, Leung KY, Tang CS (2019). Course, risk factors, and adverse outcomes of disordered eating in pregnancy. Int J Eat Disord.

[CR11] Clausen L, Rosenvinge JH, Friborg O, Rokkedal K (2011) Validating the eating disorder inventory-3 (EDI-3): a comparison between 561 female eating disorders patients and 878 females from the general population. J Psychopathol Behav Assess 33(1):101–110. 10.1007/s10862-010-9207-4 10.1007/s10862-010-9207-4 PMC3044826 21472023

[CR12] Claydon EA, Davidov DM, Zullig KJ, Lilly CL, Cottrell L, Zerwas SC (2018). Waking up every day in a body that is not yours: a qualitative research inquiry into the intersection between eating disorders and pregnancy. BMC Pregnancy Childbirth.

[CR13] Difallah D, Filatova E, Ipeirotis P (2018) Demographics and dynamics of mechanical Turk workers. In Proceedings of the Eleventh ACM International Conference on Web Search and Data Mining (WSDM ’18). Association for computing machinery, New York, NY, US. 135–143. 10.1145/3159661

[CR14] Dörsam AF, Preißl H, Micali N, Lörcher SB, Zipfel S, Giel KE (2019). The impact of maternal eating disorders on dietary intake and eating patterns during pregnancy: a systematic review. Nutrients.

[CR15] Easter A, Bye A, Taborelli E, Corfield F, Schmidt U, Treasure J, Micali N (2013). Recognising the symptoms: how common are eating disorders in pregnancy?. Eur Eat Disord Rev.

[CR16] Emery RL, Grace JL, Kolko RP, Levine MD (2017). Adapting the eating disorder examination for use during pregnancy: preliminary results from a community sample of women with overweight and obesity. Int J Eat Disord.

[CR17] Fairburn CG (2008) Eating disorder examination questionnaire (EDE-Q 6.0). In: Cognitive behavior therapy and eating disorders (pp. 309–313). New York: Guilford Press

[CR18] Hoffman DJ, Reynolds RM, Hardy DB (2017). Developmental origins of health and disease: current knowledge and potential mechanisms. Nutr Rev.

[CR19] Hubin-Gayte M, Squires C (2012). Étude de l’impact de la grossesse sur les comportements alimentaires à travers l’utilisation du questionnaire SCOFF. L’evolution Psychiatrique.

[CR20] Kennedy R, Clifford S, Burleigh T, Waggoner P, Jewell R, Winter N (2020). The shape of and solutions to the MTurk quality crisis. Polit Sci Res Methods.

[CR21] Keski-Rahkonen A, Sihvola E, Raevuori A, Kaukoranta J, Bulik CM, Hoek HW, Rissanen A, Kaprio J (2006). Reliability of self-reported eating disorders: optimizing population screening. Int J Eat Disord.

[CR22] Kimmel MC, Ferguson EH, Zerwas S, Bulik CM, Meltzer-Brody S (2015). Obstetric and gynecologic problems associated with eating disorders. Int J Eat Disord.

[CR23] Zerwas S, Claydon E (2014). Eating disorders across the lifespan: from menstruation to menopause. In D. L. Barnes (Ed.), Women's reproductive mental health across the lifespan (pp. 237–261). Springer International Publishing. 10.1007/978-3-319-05116-1_13

[CR24] Kimmel MC, Ferguson EH, Zerwas S, Bulik CM, Meltzer-Brody S (2016) Obstetric and gynecologic problems associated with eating disorders. nt J Eat Disord 49(3):260–275. 10.1002/eat.22483 10.1002/eat.22483 PMC5683401 26711005

[CR25] Leddy MA, Jones C, Morgan MA, Schulkin J (2009). Eating disorders and obstetric-gynecologic care. J Womens Health.

[CR26] Linna MS, Raevuori A, Haukka J, Suvisaari JM, Suokas JT, Gissler M (2014) Pregnancy, obstetric, and perinatal health outcomes in eating disorders. American Journal of Obstetrics and Gynecology 211(4):392-el-392.e8. 10.1016/j.ajog.2014.03.067 10.1016/j.ajog.2014.03.067 24705128

[CR27] Litman L, Robinson J (2021) Conducting online research on Amazon Mechanical Turk and beyond. Sage Publications

[CR28] Louise J, Deussen AR, Dodd JM (2020). Gestational weight gain-re-examining the current paradigm. Nutrients.

[CR29] Marchesini G, Cuzzolaro M, Mannucci E, Grave RD, Gennaro M, Tomasi F, Barantani EG, Melchionda N (2004). Weight cycling in treatment-seeking obese persons: data from the Quovadis Study. Int J Obes.

[CR30] Mahr F, Farahmand P, Bixler EO, Domen RE, Moser EM, Nadeem T, Levine RL, Halmi KA (2014). A national survey of eating disorder training. Int J Eat Disord.

[CR31] Marquer C, Barry C, Mouchenik Y, Hustache S, Djibo DM, Manzo ML, Falissard B, Révah-Lévy A, Grais RF, Moro M.-R (2012) A rapid screening tool for psychological distress in children 3–6years old: results of a validation study. BMC Psychiatry 12(170). 10.1186/1471-244x-12-170 10.1186/1471-244X-12-170 PMC3503833 23072651

[CR32] Micali N, Treasure J, Simonoff E (2007). Eating disorders symptoms in pregnancy: a longitudinal study of women with recent and past eating disorders and obesity. J Psychosom Res.

[CR33] Morgan JF, Reid F, Lacey JH (1999). The SCOFF questionnaire: assessment of a new screening tool for eating disorders. BMJ.

[CR34] Moss AJ, Rosenzweig C, Robinson J, Litman L (2020). Demographic stability on mechanical Turk despite COVID-19. Trends Cogn Sci.

[CR35] Nayak MSDP, Narayan KA (2019) Strengths and weaknesses of online sureys. IOSR Journal of Humanities and Social Sciences (IOSR-JHSS) 5(5):31–38. 10.9790/0837-2405053138

[CR36] Paslakis G, Zwaan M (2019). Clinical management of females seeking fertility treatment and of pregnant females with eating disorders. Eur Eat Disord Rev.

[CR37] Weissman RS, Becker AE, Bulik CM, Frank GKW, Klump KL, Steiger H, Strober M, Thomas J, Waller G, Walsh BT (2016). Speaking of that: terms to avoid or reconsider in the eating disorders field. Int J Eat Disord.

[CR38] Pettersson CB, Zandian M, Clinton D (2016). Eating disorder symptoms pre- and postpartum. Arch Womens Ment Health.

[CR39] SAS (2013) Statistical analysis software. Users’ guide statistics version 9.4. SAS Institute Inc., Cary

[CR40] Sebastiani G, Andreu-Fernández V, HerranzBarbero A, Aldecoa-Bilbao V, Miracle X, MelerBarrabes E, Balada Ibañez A, Astals-Vizcaino M, Ferrero-Martínez S, Gómez-Roig MD, García-Algar O (2020). Eating disorders during gestation: implications for mother’s health, fetal outcomes, and epigenetic changes. Front Pediatr.

[CR41] Soares RM, Nunes MA, Schmidt MI, Giacomello A, Manzolli P, Camey S, Buss C, Drehmer M, Melere C, Hoffman J, Ozcariz S, Manenti CN, Pinheiro AP, Duncan BB (2009) Inappropriate eating behaviors during pregnancy: prevalence and associated factors among pregnant women attending primary care in southern Brazil. Int J Eat Disord 42(5):387–393. 10.1002/eat.20643 10.1002/eat.20643 19115363

[CR42] Watson H, Von Holle A, Hamer R, Knoph Berg C, Torgersen L, Magnus P, Bulik C (2013) Remission, continuation and incidence of eating disorders during early pregnancy: a validation study in a population-based birth cohort. Psychol Med 43(8):1723–1734. 10.1017/S0033291712002516 10.1017/S0033291712002516 PMC4206832 23164164

[CR43] Streiner DL (2003). Starting at the beginning: an introduction to coefficient alpha and internal consistency. J Pers Assess.

[CR44] Sysko R, Glasofer DR, Hildebrandt T, Klimek P, Mitchell JE, Berg KC, Peterson CB, Wonderlich SA, Walsh BT (2015). The eating disorder assessment for DSM-5 (EDA-5): development and validation of a structured interview for feeding and eating disorders. Int J Eat Disord.

