# Supplementary material for: Development and validation across trimester of the Prenatal Eating Behaviors Screening tool
Source: Arch Womens Ment Health. 2022 May 2;25(4):705–16. doi: 10.1007/s00737-022-01230-y (PMC9058752; doi:10.1007/s00737-022-01230-y)
Supplement: Supplementary file 1 — Supplementary file1 (DOCX 21 KB) [file 737_2022_1230_MOESM1_ESM.docx]

| **Item** | **Development** | | | | | **Validation** | | | | |
| --- | --- | --- | --- | --- | --- | --- | --- | --- | --- | --- |
|  | **Very Uncomfortable N(%)** | **Somewhat Uncomfortable N(%)** | **Neither Comfortable nor Uncomfortable N(%)** | **Somewhat Comfortable N(%)** | **Very Comfortable N(%)** | **Very Uncomfortable N(%)** | **Somewhat Uncomfortable N(%)** | **Neither Comfortable nor Uncomfortable N(%)** | **Somewhat Comfortable N(%)** | **Very Comfortable N(%)** |
| How Comfortable are you with Being Weighed Today by a Health Professional? | 10 (5.26%) | 22 (11.58%) | 14 (7.37%) | 81 (42.63%) | 63 (33.16%) | 7 (4.19%) | 31 (18.56%) | 12 (7.19%) | 63 (37.72%) | 54 (32.34%) |
|  | **Very Dissatisfied N(%)** | **Dissatisfied N(%)** | **Neither Satisfied or Dissatisfied N(%)** | **Satisfied N(%)** | **Very Satisfied N(%)** | **Very Dissatisfied N(%)** | **Dissatisfied N(%)** | **Neither Satisfied or Dissatisfied N(%)** | **Ssatisfied N(%)** | **Very Satisfied N(%)** |
| Are you Satisfied with your Pregnancy Weight Gain? | 8 (4.21%) | 22 (11.58%) | 37 (19.47%) | 97 (51.05%) | 26 (13.68%) | 7 (4.19%) | 37 (22.16%) | 48 (38.74%) | 53 (31.74%) | 22 (13.17%) |
| During this Pregnancy, how Satisfied are you with your Weight Progression? | 5 (2.63%) | 27 (14.21%) | 46 (24.21%) | 80 (42.11%) | 32 (16.84%) | 8 (4.79%) | 32 (19.16%) | 41 (24.55%) | 67 (40.12%) | 19 (11.38%) |
| During this Pregnancy, how Satisfied are you Currently Seeing your own body in a mirror, while undressing, etc.? | 13 (6.84%) | 39 (20.53%) | 33 (18.37%) | 69 (36.32%) | 36 (18.95%) | 17 (10.18%) | 40 (23.95%) | 39 (23.35%) | 47 (28.14%) | 24 (14.37%) |
| During this Pregnancy, How Satisfied are you with your Body's Appearance Generally? | 16 (8.42%) | 35 (18.42%) | 32 (16.84%) | 81 (42.63%) | 26 (13.68%) | 12 (7.19%) | 38 (22.75%) | 34 (20.36%) | 67 (40.12%) | 16 (9.58%) |
|  | **Never N(%)** | **Rarely (e.g., once or twice) N(%)** | **Occasionally (e.g., every few weeks/monthly) N(%)** | **A Moderate Amount (e.g., weekly) N(%)** | **A Great Deal (e.g., daily) N(%)** | **Never N(%)** | **Rarely (e.g., once or twice) N(%)** | **Occasionally (e.g., every few weeks/monthly) N(%)** | **A Moderate Amount (e.g., weekly) N(%)** | **A Great Deal (e.g., daily) N(%)** |
| During this pregnancy, how frequently, if at all, have you used any pregnancy symptoms to control weight? (e.g., morning sickness, nausea, etc.) | 58 (30.53%) | 26 (13.68%) | 29 (15.26%) | 53 (27.89%) | 24 (12.63%) | 52 (31.14%) | 33 (19.76%) | 23 (13.77%) | 45 (26.95%) | 14 (8.38%) |
| During this pregnancy, how frequently, if at all, have you used diuretics, laxatives, or detox supplements to control your weight or shape in response to food intake? (e.g., probiotics, metabolism boosters, Lasix, etc.) | 96 (50.53%) | 11 (5.79%) | 22 (11.58%) | 39 (20.53%) | 22 (11.58%) | 96 (57.49%) | 17 (10.18%) | 24 (14.37%) | 24 (14.37%) | 6 (3.59%) |
| During this pregnancy, how frequently, if at all, have you made yourself sick after eating in order to control your weight or shape? | 97 (51.05%) | 12 (6.32%) | 20 (10.53%) | 39 (20.53%) | 22 (11.58%) | 109 (65.27%) | 19 (11.38%) | 21 (12.57%) | 11 (6.59%) | 7 (4.19%) |
| During this pregnancy, how frequently, if at all, did you excessively exercise as a response to food intake? (e.g., to influence weight or shape) | 77 (40.53%) | 22 (11.58%) | 26 (13.68%) | 40 (21.05%) | 25 (13.16%) | 78 (46.71%) | 33 (19.76%) | 28 (16.77%) | 17 (10.18%) | 11 (6.59%) |
| During this pregnancy, how frequently, if at all, did you avoid eating any foods which you like in order to influence your shape or weight? | 39 (20.53%) | 27 (14.21%) | 30 (15.79%) | 64 (33.68%) | 30 (15.79%) | 51 (30.54%) | 28 (16.77%) | 37 (22.16%) | 34 (20.36%) | 17 (10.18%) |
| During this pregnancy, how frequently, if at all, did you think of trying to vomit in order to lose weight? | 86 (45.26%) | 24 (12.63%) | 26 (13.68%) | 27 (14.21%) | 27 (14.21%) | 114 (68.26%) | 14 (8.38%) | 13 (7.78%) | 17 (10.18%) | 9 (5.39%) |
| During this pregnancy, how frequently, if at all, did you experience a loss of control in overeating unrelated to pregnancy cravings? | 59 (31.05%) | 25 (13.16%) | 35 (18.42%) | 51 (26.84%) | 20 (10.53%) | 47 (28.14%) | 31 (18.56%) | 47 (28.14%) | 28 (16.77%) | 14 (8.38%) |
| During this pregnancy, how frequently, if at all, did you go on eating binges where you felt that you could not stop? | 62 (32.63%) | 27 (14.21%) | 43 (22.63%) | 40 (21.05%) | 18 (9.47%) | 47 (28.14%) | 32 (19.16%) | 43 (25.75%) | 31 (18.56%) | 14 (8.38%) |
| During this pregnancy, how frequently, if at all, did you spend a majority of your day thinking about food, weight, counting calories, or other weight related topics? | 36 (18.95%) | 28 (14.74%) | 44 (23.16%) | 48 (25.26%) | 34 (17.89%) | 38 (22.75%) | 35 (20.96%) | 48 (28.74%) | 32 (19.16%) | 14 (8.38%) |
| During this pregnancy, how frequently, if at all, did you feel you couldn't control what you were eating and/or excessively exercise in order to control your weight? | 61 (32.11%) | 25 (13.16%) | 35 (18.42%) | 49 (25.79%) | 20 (10.53%) | 53 (31.74%) | 40 (23.95%) | 37 (22.16%) | 2o (11.98%) | 17 (10.18%) |
| During this pregnancy, how frequently, if at all, did you emphasize the importance of weight? (e.g., to others, to yourself, etc.) | 23 (12.11%) | 32 (16.84%) | 48 (25.26%) | 52 (27.37%) | 35 (18.42%) | 33 (19.76%) | 31 (18.56%) | 56 (33.53%) | 24 (14.37%) | 23 (13.77%) |
| During your pregnancy, how frequently, if at all, has thinking about your shape or weight interfered with your ability to concentrate on things? | 46 (24.21%) | 30 (15.79%) | 34 (17.89%) | 55 (28.95%) | 25 (13.16%) | 49 (29.34%) | 34 (20.36%) | 43 (25.75%) | 26 (15.57%) | 15 (8.98%) |
| During this pregnancy, how frequently, if at all, have you felt guilty about eating? | 33 (17.37%) | 33 (17.37%) | 47 (24.74%) | 46 (24.21%) | 31 (16.32%) | 41 (24.55%) | 32 (19.16%) | 37 (22.16%) | 35 (20.96%) | 22 (13.17%) |
| During your pregnancy, how frequently, if at all, have you restricted your portion sizes? | 39 (20.53%) | 35 (18.42%) | 44 (23.16%) | 44 (23.16%) | 28 (14.74%) | 42 (25.15%) | 43 (25.75%) | 40 (23.95%) | 31 (18.56%) | 11 (6.59%) |
|  | **Strongly Disagree N(%)** | **Disagree N(%)** | **Neither Agree nor Disagree N(%)** | **Agree N(%)** | **Strongly Agree N(%)** | **Strongly Disagree N(%)** | **Disagree N(%)** | **Neither Agree nor Disagree N(%)** | **Agree N(%)** | **Strongly Agree N(%)** |
| During this pregnancy, you eat much more when you are alone than when you are in front of others. | 23 (12.11%) | 32 (16.84%) | 24 (12.63%) | 76 (40.00%) | 35 (18.42%) | 27 (16.17%) | 36 (21.56%) | 28 (16.77%) | 49 (29.34%) | 27 (16.17%) |
| During this pregnancy, food has dominated your life. | 22 (11.58%) | 35 (18.42%) | 37 (19.47%) | 63 (33.16%) | 33 (17.37%) | 29 (17.37%) | 40 (23.95%) | 39 (23.35%) | 43 (25.75%) | 16 (9.58%) |
| During this pregnancy, you have had the desire for your stomach to feel hungry. | 38 (20.00%) | 32 (16.84%) | 28 (14.74%) | 63 (33.16%) | 29 (15.26%) | 39 (23.35%) | 38 (22.75%) | 33 (19.76%) | 40 (23.95%) | 17 (10.18%) |
| During this pregnancy, you have been fearful of weight gain. | 17 (8.95%) | 33 (17.37%) | 27 (14.21%) | 74 (38.95%) | 39 (20.53%) | 23 (13.77%) | 27 (16.17%) | 30 (17.96%) | 60 (35.93%) | 27 (16.17%) |
| During this pregnancy, you have been fearful of losing your pregnancy weight. | 25 (13.16%) | 35 (18.42%) | 29 (15.26%) | 64 (33.68%) | 37 (19.47%) | 26 (15.57%) | 41 (24.55%) | 26 (15.57%) | 54 (32.34%) | 20 (11.98%) |
| During this pregnancy, it bothers you when you are weighed (e.g., at the doctor's office.) | 19 (10.00%) | 38 (20.00%) | 30 (15.79%) | 73 (38.42%) | 30 (15.79%) | 32 (19.16%) | 38 (22.75%) | 31 (18.56%) | 46 (27.54%) | 20 (11.98%) |
