# Supplementary material for: Development and validation across trimester of the Prenatal Eating Behaviors Screening tool
Source: Arch Womens Ment Health. 2022 May 2;25(4):705–16. doi: 10.1007/s00737-022-01230-y (PMC9058752; doi:10.1007/s00737-022-01230-y)
Supplement: Supplementary file 2 — Supplementary file2 (DOCX 36 KB) [file 737_2022_1230_MOESM2_ESM.docx]

Eating Behaviors & Attitudes During Pregnancy Survey

*Dear Participant, 
This letter is a request for you to take part in a research study about eating behaviors and attitudes towards weight during pregnancy. This project is being conducted by Dr. Elizabeth Claydon in the Department of Social & Behavioral Sciences at WVU School of Public Health. Your participation in this project is greatly appreciated and will take approximately 5 minutes to fill out the attached questionnaire. Your involvement in this project will be kept as confidential as legally possible. All data will be reported in the aggregate. You must be 18 years of age or older to participate. I will not ask any information that should lead back to your identity as a participant. Your participation is completely voluntary. You may skip any question that you do not wish to answer and you may discontinue at any time. West Virginia University's Institutional Review Board acknowledgment of this project is on file. 
Thank you very much for your time. Should you have any questions about this letter or the research project, please feel free to contact Dr. Claydon at (304) 293-1900 or by e-mail at elizabeth.claydon@hsc.wvu.edu.*
 *Thank you for your time and help with this project. 
Sincerely,*

*Elizabeth Claydon, PhD, MPH, MS*

*Assistant Professor*

*Social & Behavioral Sciences*

*West Virginia University*

I agree to participate in this study.

- Yes (1)
- No (2)

Skip To: End of Survey If I agree to participate in this study. = No

| 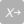 |
| --- |

What is your current age?

▼ Under 18 (1) ... 55 - 64 (6)

What is your relationship status?

- Married (1)
- Widowed (2)
- Divorced (3)
- Separated (4)
- Single (5)
- Living with a Partner (6)
- In a Relationship (7)

| 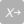 |
| --- |

What race do you consider yourself to be?

- White (1)
- Black or African American (2)
- American Indian or Alaska Native (3)
- Asian (4)
- Native Hawaiian or Pacific Islander (5)
- Other (6)

Are you of Hispanic of Latino descent?

- Yes (1)
- No (2)

| 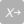 |
| --- |

How many children do you have?

▼ 0 (1) ... 5+ (6)

| 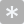 |
| --- |

How many pregnancies including this one have you had?

________________________________________________________________

| 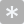 |
| --- |

What week of your current pregnancy are you in?

________________________________________________________________

| 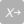 |
| --- |

What is your average total household annual income?

- Less than 19k (1)
- 20k-49k (2)
- 50-149k (3)
- 150-249k (4)
- 250k+ (5)
- Prefer not to answer (6)

| 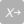 |
| --- |

What is your working status?

- Student (either unemployed or employed part-time) (1)
- Unemployed or seeking employment (2)
- Retired/disability (3)
- Part-time (4)
- Full-time (5)
- Prefer not to answer (6)

What medical insurance do you have?

- Private (1)
- State (eg, Medicaid) (2)
- None (3)
- Prefer not to answer (4)

Have you ever been diagnosed with an eating disorder?

- Yes (1)
- No (2)

Skip To: Ht If Have you ever been diagnosed with an eating disorder? = No

| 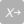 |
| --- |

Which eating disorders have you had? (Select as many as apply.)

|  | Current Professional Diagnosis (1) | Past Professional Diagnosis (2) | Current Self Diagnosis (3) | Past Self Diagnosis (4) |
| --- | --- | --- | --- | --- |
| Anorexia Nervosa (AN) |  |  |  |  |
| Bulimia Nervosa (BN) |  |  |  |  |
| Binge Eating Disorder (BED) |  |  |  |  |
| OSFED formerly EDNOS |  |  |  |  |

What was the duration of your eating disorder (if you experienced a relapse, choose the best fitting category)

- <1 year (1)
- 1-3 years (2)
- 4-6 years (3)
- 7-10 years (4)
- 10+ years (5)

Are you currently seeking treatment or counseling for an eating disorder?

- I am seeking counseling (1)
- I am seeking in-patient treatment (2)
- I am not seeking counseling or treatment (3)

What is your current height? (if you do not feel comfortable answering this, please skip to the next question)

- Feet (15) ________________________________________________
- Inches (17) ________________________________________________

If you are comfortable answering, what was your pre-pregnancy weight?

________________________________________________________________

If you are comfortable answering, what has been your weight change during this pregnancy?

▼ Lost more than 15 pounds (1) ... Gained 70 to 79 pounds (14)

What is the highest year of school or college that you have completed?

- Grade School (1)
- High School/GED (2)
- Vocational School (3)
- College (4)
- Professional/Graduate School (5)

End of Block: Demographic Questions

Start of Block: Eating Behavior Questions

How comfortable are you with being weighed today by a health professional?

- Very Comfortable (1)
- Somewhat comfortable (2)
- Neither comfortable nor uncomfortable (3)
- Somewhat uncomfortable (4)
- Not comfortable at all (5)

| 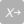 |
| --- |

Are you satisfied with your pregnancy weight gain?

- Very satisfied (1)
- Satisfied (2)
- Neither satisfied nor dissatisfied (3)
- Dissatisfied (4)
- Very dissatisfied (5)

| 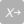 |
| --- |

During this pregnancy, how satisfied are you with your weight progession?

- Very satisfied (1)
- Satisfied (2)
- Neither satisfied nor dissatisfied (3)
- Dissatisfied (4)
- Very dissatisfied (5)

| 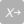 |
| --- |

During this pregnancy, how satisfied are you currently seeing your own body in a mirror, while undressing, etc.?

- Very satisfied (1)
- Satisfied (2)
- Neither satisfied nor dissatisfied (3)
- Dissatisfied (4)
- Very dissatisfied (5)

| 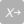 |
| --- |

During this pregnancy, how satisfied are you with your body's appearance generally?

- Very satisfied (1)
- Satisfied (2)
- Neither satisfied nor dissatisfied (3)
- Dissatisfied (4)
- Very dissatisfied (5)

| 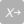 |
| --- |

During this pregnancy, how frequently, if at all, have you used any pregnancy symptoms to control weight? (e.g., morning sickness, nausea, etc.)

- A great deal (e.g., daily) (5)
- A moderate amount (e.g., weekly) (4)
- Occasionally (e.g., every few weeks/monthly) (3)
- Rarely (e.g., once or twice) (2)
- Never (1)

| 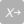 |
| --- |

During this pregnancy, how frequently, if at all, have you used diuretics, laxatives, or detox supplements to control your weight or shape in response to food intake? (e.g., probiotics, metabolism boosters, Lasix, etc.)

- A great deal (e.g., daily) (5)
- A moderate amount (e.g., weekly) (4)
- Occasionally (e.g., every few weeks/monthly) (3)
- Rarely (e.g., once or twice) (2)
- Never (1)

| 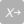 |
| --- |

During this pregnancy, how frequently, if at all, have you made yourself sick after eating in order to control your weight or shape?

- A great deal (e.g., daily) (5)
- A moderate amount (e.g., weekly) (4)
- Occasionally (e.g., every few weeks/monthly) (3)
- Rarely (e.g., once or twice) (2)
- Never (1)

| 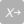 |
| --- |

During this pregnancy, how frequently, if at all, did you excessively exercise as a response to food intake? (e.g., to influence weight or shape)

- A great deal (e.g., daily) (5)
- A moderate amount (e.g., weekly) (4)
- Occasionally (e.g., every few weeks/monthly) (3)
- Rarely (e.g., once or twice) (2)
- Never (1)

| 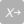 |
| --- |

During this pregnancy, how frequently, if at all, did you avoid eating any foods which you like in order to influence your shape or weight?

- A great deal (e.g., daily) (5)
- A moderate amount (e.g., weekly) (4)
- Occasionally (e.g., every few weeks/monthly) (3)
- Rarely (e.g., once or twice) (2)
- Never (1)

| 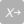 |
| --- |

During this pregnancy, how frequently, if at all, did you think of trying to vomit in order to lose weight?

- A great deal (e.g., daily) (5)
- A moderate amount (e.g., weekly) (4)
- Occasionally (e.g., every few weeks/monthly) (3)
- Rarely (e.g., once or twice) (2)
- Never (1)

| 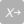 |
| --- |

During this pregnancy, how frequently, if at all, did you experience a loss of control in overeating unrelated to pregnancy cravings?

- A great deal (e.g., daily) (5)
- A moderate amount (e.g., weekly) (4)
- Occasionally (e.g., every few weeks/monthly) (3)
- Rarely (e.g., once or twice) (2)
- Never (1)

| 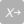 |
| --- |

During this pregnancy, how frequently, if at all, did you go on eating binges where you felt that you could not stop?

- A great deal (e.g., daily) (5)
- A moderate amount (e.g., weekly) (4)
- Occasionally (e.g., every few weeks/monthly) (3)
- Rarely (e.g., once or twice) (2)
- Never (1)

| 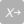 |
| --- |

During this pregnancy, how frequently, if at all, did you spend a majority of your day thinking about food, weight, counting calories, or other weight related topics?

- A great deal (e.g., daily) (5)
- A moderate amount (e.g., weekly) (4)
- Occasionally (e.g., every few weeks/monthly) (3)
- Rarely (e.g., once or twice) (2)
- Never (1)

| 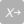 |
| --- |

During this pregnancy, how frequently, if at all, did you feel you couldn't control what you were eating and/or excessively exercise in order to control your weight?

- A great deal (e.g., daily) (5)
- A moderate amount (e.g., weekly) (4)
- Occasionally (e.g., every few weeks/monthly) (3)
- Rarely (e.g., once or twice) (2)
- Never (1)

| 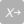 |
| --- |

During this pregnancy, how frequently, if at all, did you emphasize the importance of weight? (e.g., to others, to yourself, etc.)

- A great deal (e.g., daily) (5)
- A moderate amount (e.g., weekly) (4)
- Occasionally (e.g., every few weeks/monthly) (3)
- Rarely (e.g., once or twice) (2)
- Never (1)

| 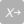 |
| --- |

During your pregnancy, how frequently, if at all, has thinking about your shape or weight interfered with your ability to concentrate on things?

- A great deal (e.g., daily) (5)
- A moderate amount (e.g., weekly) (4)
- Occasionally (e.g., every few weeks/monthly) (3)
- Rarely (e.g., once or twice) (2)
- Never (1)

| 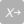 |
| --- |

During this pregnancy, how frequently, if at all, have you felt guilty about eating?

- A great deal (e.g., daily) (5)
- A moderate amount (e.g., weekly) (4)
- Occasionally (e.g., every few weeks/monthly) (3)
- Rarely (e.g., once or twice) (2)
- Never (1)

| 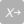 |
| --- |

During your pregnancy, how frequently, if at all, have you restricted your portion sizes?

- A great deal (e.g., daily) (5)
- A moderate amount (e.g., weekly) (4)
- Occasionally (e.g., every few weeks/monthly) (3)
- Rarely (e.g., once or twice) (2)
- Never (1)

| 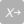 |
| --- |

During this pregnancy, you eat much more when you are alone than when you are in front of others.

- Strongly agree (5)
- Agree (4)
- Neither agree nor disagree (3)
- Disagree (2)
- Strongly disagree (1)

| 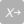 |
| --- |

During this pregnancy, food has dominated your life.

- Strongly agree (5)
- Agree (4)
- Neither agree nor disagree (3)
- Disagree (2)
- Strongly disagree (1)

| 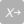 |
| --- |

During this pregnancy, you have had the desire for your stomach to feel hungry.

- Strongly agree (5)
- Agree (4)
- Neither agree nor disagree (3)
- Disagree (2)
- Strongly disagree (1)

| 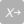 |
| --- |

During this pregnancy, you have been fearful of weight gain.

- Strongly agree (5)
- Agree (4)
- Neither agree nor disagree (3)
- Disagree (2)
- Strongly disagree (1)

| 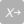 |
| --- |

During this pregnancy, you have been fearful of losing your pregnancy weight.

- Strongly agree (5)
- Agree (4)
- Neither agree nor disagree (3)
- Disagree (2)
- Strongly disagree (1)

| 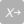 |
| --- |

During this pregnancy, it bothers you when you are weighed (e.g., at the doctor's office.)

- Strongly agree (5)
- Agree (4)
- Neither agree nor disagree (3)
- Disagree (2)
- Strongly disagree (1)

REFERRAL LIST
Below is a list of Referrals, Helplines, & Support Groups

Inclusion on the list does not imply the WVU School of Public Health’s endorsement of these agencies or services. Payment for services rendered at these agencies is the sole responsibility of the patient and not the researchers of the current study.         
**Referrals Academy for Eating Disorders: Find a Professional**

<http://www.aedweb.org/index.php/education/eating-disorder-information-2>

**Helplines**

National Suicide Prevention Lifeline **1-800-273-TALK**or **1-800-SUICIDE (784-2433)**

**National Eating Disorder Association (NEDA) free, confidential Helpline**

Monday – Thursday 9am-9pm, Friday 9am-5pm**1-800-931-2237**

Substance Abuse and Mental Health Services Administration (SAMHSA) Helpline**1-800-662-HELP (4357)**
