# Supplementary material for: Development and validation across trimester of the Prenatal Eating Behaviors Screening tool
Source: Arch Womens Ment Health. 2022 May 2;25(4):705–16. doi: 10.1007/s00737-022-01230-y (PMC9058752; doi:10.1007/s00737-022-01230-y)
Supplement: Supplementary file 3 — Supplementary file3 (PDF 3305 KB) [file 737_2022_1230_MOESM3_ESM.pdf]

WEST VIRGINIA UNIVERSITY

EATING BEHAVIORS DURING PREGNANCY RESEARCH SURVEY (IRB: 2003925385)

Volunteers to Complete Quick Research Survey

Eligible participants are asked to complete a short (5-10 minute questionnaire) about their eating behaviors and attitudes towards weight during pregnancy. This questionnaire can be completed while waiting for or after a routine prenatal appointment. All information will be kept confidential.

https://bit.ly/3ggAdP4

Paper Copies can be found at the reception desk.

Eligibility criteria include:
Women (age 18 and older) that are currently pregnant.

WVU IRB acknowledgment of the study is on file

If any further questions regarding the study, please contact Dr. Elizabeth Claydon:
elizabeth.claydon@hsc.wvu.edu
