# Supplementary figures and images for: Development and validation across trimester of the Prenatal Eating Behaviors Screening tool
Source: Arch Womens Ment Health. 2022 May 2;25(4):705–16. doi: 10.1007/s00737-022-01230-y (PMC9058752; doi:10.1007/s00737-022-01230-y)

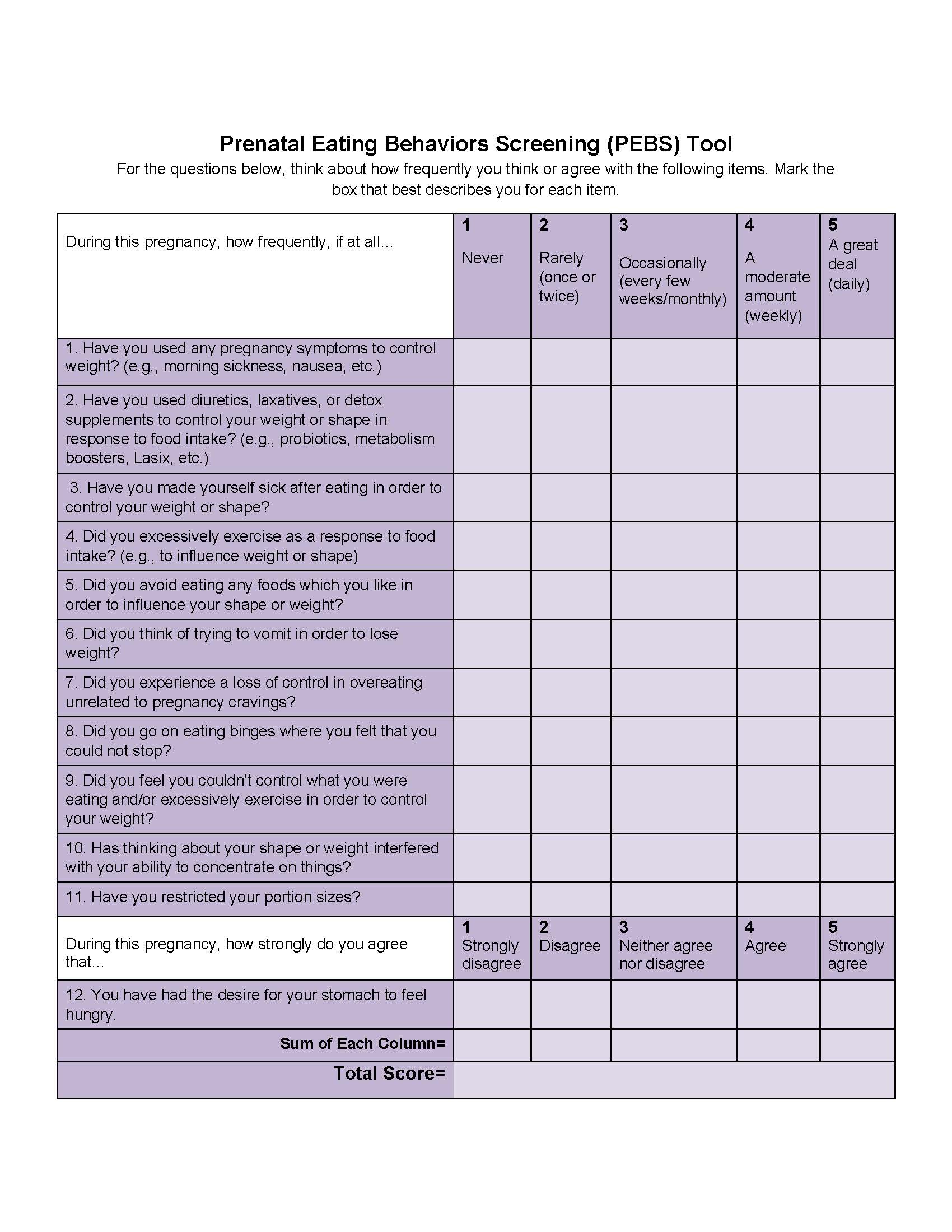

Supplement: Supplementary file 4 — Supplementary file4 (JPG 293 KB) [file 737_2022_1230_MOESM4_ESM.jpg]
