# Supplementary material for: Development and validation across trimester of the Prenatal Eating Behaviors Screening tool
Source: Arch Womens Ment Health. 2022 May 2;25(4):705–16. doi: 10.1007/s00737-022-01230-y (PMC9058752; doi:10.1007/s00737-022-01230-y)
Supplement: Supplementary file 5 — Supplementary file5 (PDF 166 KB) [file 737_2022_1230_MOESM5_ESM.pdf]

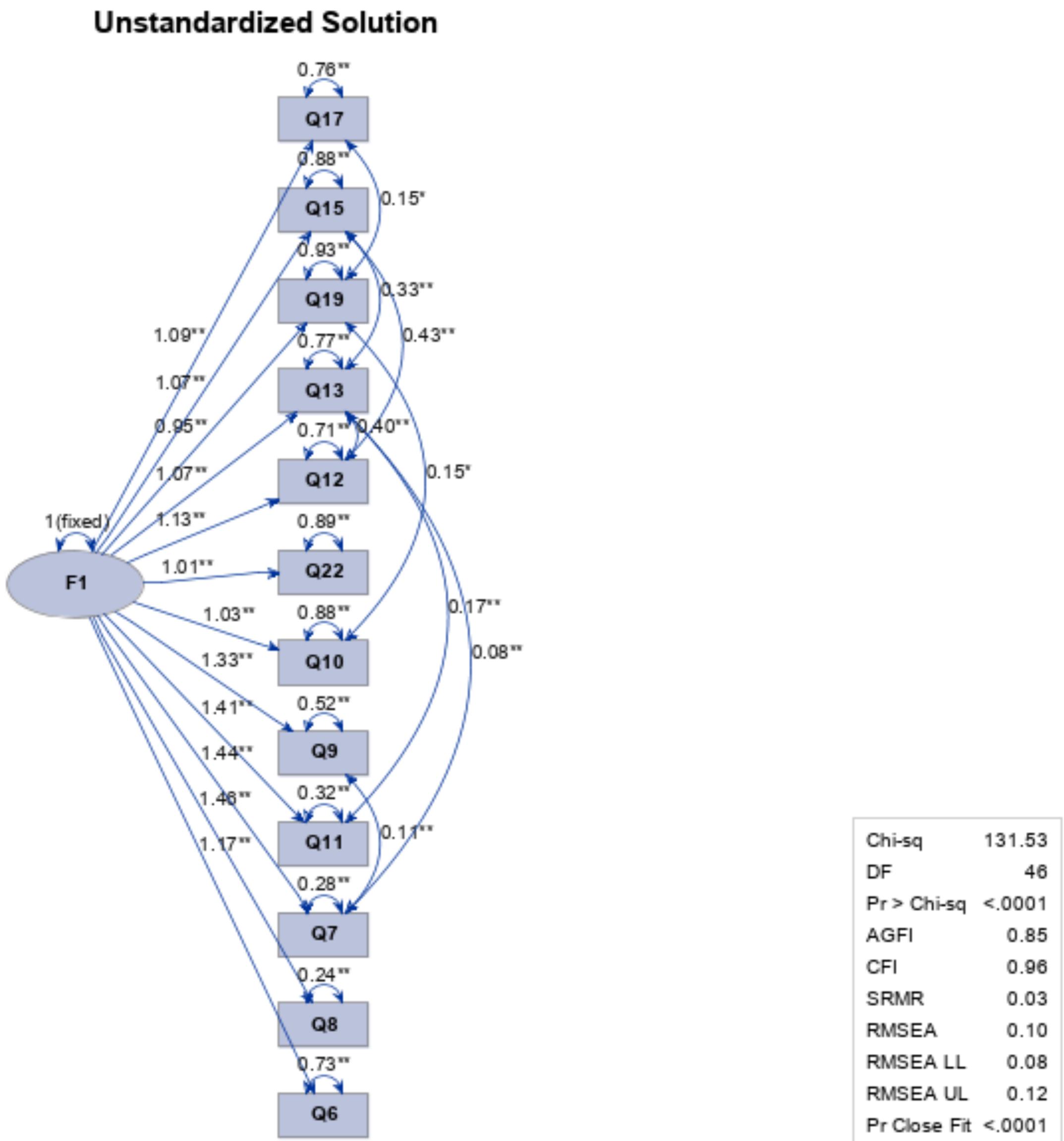


**Fig. A1 Path Diagram for Factor Loading**
